# Comprehensive assessment of tissue and serum parameters of bone metabolism in a series of orthopaedic patients

**DOI:** 10.1371/journal.pone.0227133

**Published:** 2019-12-27

**Authors:** Jan Gunsser, Regina Hermann, Andreas Roth, Amelie Lupp

**Affiliations:** 1 Institute of Pharmacology and Toxicology, Jena University Hospital, Jena, Germany; 2 Department of Internal Medicine 2, HELIOS Hospital Erfurt, Erfurt, Germany; 3 Orthopaedic Professorship of the University Hospital Jena, Orthopaedic Department of the Waldkliniken, former Rudolf Elle Hospital, Eisenberg, Germany; 4 Department of Orthopaedics, Traumatology and Plastic Surgery, Division of Endoprosthetics/Orthopaedics, University Hospital Leipzig, Leipzig, Germany; Texas A&M University College of Dentistry, UNITED STATES

## Abstract

Bone diseases represent an increasing health burden worldwide, and basic research remains necessary to better understand the complexity of these pathologies and to improve and expand existing prevention and treatment approaches. In the present study, 216 bone samples from the *caput femoris* and *collum femoris* of 108 patients with degenerative or dysplastic coxarthrosis, hip fracture, or osteonecrosis were evaluated for the proportion of trabecular bone (TB) and expression of parathyroid hormone (PTH) type 1 receptor (PTH1R), osteoprotegerin (OPG), and receptor activator of nuclear factor kappa-B ligand (RANKL). Serum levels of PTH, OPG, soluble RANKL (sRANKL), alkaline phosphatase (AP), osteocalcin, total procollagen type-1 intact N-terminal propeptide (TP1NP), tartrate-resistant acid phosphatase type 5b (TRAP5b), sclerostin, and C-telopeptide of type-1 collagen (ICTP) were also determined. Age was positively correlated with serum levels of PTH, OPG, and sclerostin but negatively associated with TB and sRANKL. Women exhibited less TB, lower sclerostin and ICTP, and higher TRAP5b. Impaired kidney function was associated with shorter bone decalcification time, less TB, lower sRANKL, and higher serum PTH, OPG, and sclerostin. Furthermore, correlations were observed between bone PTH1R and OPG expression and between serum PTH, OPG, and AP. There were also positive correlations between serum OPG and TP1NP; serum OPG and sclerostin; serum AP, osteocalcin, and TRAP5b; and serum sclerostin and ICTP. Serum OPG was negatively associated with sRANKL. In summary, clear relationships between specific bone metabolism markers were observed, and distinct influences of age, sex, and kidney function, thus underscoring their suitability as diagnostic or prognostic markers.

## Introduction

Bone diseases, such as osteoporosis, represent one of the major health problems worldwide. They are especially common in the elderly but may affect people of any age [1–3]. In addition to pain and deformity, fractures are a major complication of bone disease, leading to increased morbidity, reduced quality of life, and even death. The care of patients with bone diseases is very costly to society, and expenses will continue to rise as the frequency of bone diseases increases in the future with the anticipated growth of the elderly population. Therefore, despite modern prophylaxis and treatment options, basic research efforts are still necessary to better understand the complex pathologies of different bone diseases and to improve and expand treatment approaches.

Human bone undergoes constant remodelling, which is controlled by endocrine and paracrine signals. Osteoblasts, osteocytes, and osteoclasts represent the most important types of cells in bone, and these cells are controlled by many hormones. Without osteoblasts, no osteoclasts can be formed, so excessive bone loss is prevented. Osteoclastogenesis requires macrophage colony-stimulating factor and receptor activator of nuclear factor kappa-B ligand (RANKL), which is expressed by osteoblasts, as well as RANK, which is present on osteoclast precursor cells [4, 5]. Parathyroid hormone (PTH), vitamin D3, and oestrogens may influence the expression of RANKL. In its active form, PTH is an 84-amino acid peptide hormone, which is produced in the parathyroid glands. It plays an important role in calcium and phosphate homeostasis and provides rapid mobilization of calcium. In the kidneys, PTH promotes reabsorption of calcium and inhibits phosphate reuptake. In bone, PTH indirectly activates osteoclasts, thus leading to the release of calcium. These actions of PTH are mediated via the PTH type 1 receptor (PTH1R), a plasma membrane-bound G protein-coupled receptor. Besides PTH1R another receptor exists, PTH2R, which has no importance in bone metabolism. Osteoprotegerin (OPG) is a secretory, 380-amino acid glycoprotein belonging to the family of tumour necrosis factors/tumour necrosis factor receptors. In bone, OPG is secreted by osteoblasts and prevents RANK from binding to RANKL, thereby reducing recruitment, activation, and proliferation of osteoclasts [4, 6, 7].

Although the effects of many physiological and pathophysiological factors in specific bone diseases are well known, there is currently no comprehensive evaluation of the interrelationships between the various tissue and serum parameters of bone metabolism, as well as the changes that occur with age, sex, and kidney function, especially in humans.

In the present study, we assessed a panel of serum parameters of bone metabolism − the bone formation markers PTH, OPG, alkaline phosphatase (AP), osteocalcin, and total procollagen type-1 intact N-terminal propeptide (TP1NP), the bone resorption markers tartrate-resistant acid phosphatase type 5b (TRAP5b), soluble RANKL (sRANKL), and C-telopeptide of type-1 collagen (ICTP), and sclerostin, which exerts anti-anabolic effects on bone formation, − in a consecutively recruited series of orthopaedic patients who underwent hip replacement surgery. These parameters were evaluated for possible interrelationships, and correlated with bone histomorphology; bone PTH1R, OPG, and RANKL expression; and clinical data, such as age, sex, serum calcium and kidney function.

## Materials and methods

### Bone and serum samples

A total of 216 bone samples were obtained from 108 patients who underwent hip replacement surgery in the Rudolf Elle Hospital, Eisenberg, Germany, during 2010 and 2011. The patients were recruited consecutively and thus represent the typical clientele of such a medium-sized clinic. The specimens were obtained from the *caput femoris* (samples A, containing an additional layer of cartilage and cortical bone at the upper surface; n = 108) and *collum femoris* (samples B, consisting of primarily cancellous bone; n = 108). One specimen from each location was retrieved from each patient, thus allowing for an evaluation of the effects of age, sex or kidney function on cortical and cancellous bone separately. For standardization, the sampling was always carried out by the same surgeon (AR). Two punching cylinders with a diameter of 6 mm and a length of 20 mm were removed always at the same place of the coxal femur end using a cannulated reamer (Medicon, Tuttlingen, Germany). One cylinder was removed in the main superior loading zone of the femoral head, perpendicular to the surface. After resection of the hip head in the middle of the thigh neck, the second sample was taken centrally from the marrow area of the femur in the course of the rest of the thigh neck. The bones samples were placed in 10% buffered formalin immediately after removal, where they remained for several weeks until further processing. Additionally, blood was drawn from the patients during surgery, serum was prepared according to a standardized protocol, snap-frozen and stored at –80°C until analysis. Demographic and clinical data, such as age, sex, serum calcium, serum creatinine, and glomerular filtration rate (GFR), were retrieved from the patients’ records. The indications for surgery were mostly degenerative coxarthrosis (n = 92), followed by dysplastic coxarthrosis (n = 9), hip fracture (n = 5), and osteonecrosis (n = 2). Of the 216 bone samples, 120 were from the right *caput/collum femoris* and 96 were from the left *caput/collum femoris*.

All procedures involving human participants were performed in accordance with the ethical standards of the institutional and national research committees, as well as the 1964 Helsinki Declaration and its later amendments. Permission was obtained from the local ethics committee (Ethikkommission des Universitätsklinikums Jena) for the study. Written informed consent for the use of tissue and serum samples for scientific purposes was obtained from all individual participants included in the study. All data were analyzed anonymously.

### Histological methods

The bone samples were decalcified at room temperature using an ethylene-diamine-tetraacetic acid-containing solution (Osteosoft®, Merck Millipore, Darmstadt, Germany). The solution was exchanged every 3 days. After being put in the decalcification solution, the start time was noted and the samples were daily checked for their consistency. After successful decalcification, the end time was noted again. Decalcification time varied between 1 and 8 weeks, depending on the consistency of the bone. Following decalcification, the samples were dehydrated in graded ethanol and embedded in paraffin blocks. From these blocks, 4-μm sections were prepared using a microtome (Microm HM 335 E, Microm, Walldorf, Germany) and floated onto positively-charged slides.

Sections of each sample were stained with hematoxylin and eosin (HE) according to standard laboratory protocols or by immunohistochemistry. Immunostaining was performed using an indirect peroxidase labelling method, as described previously [8]. Briefly, sections were dewaxed, microwaved in 10 mM citric acid (pH 6.0) for 16 min at 600 W, and then incubated overnight at 4°C with a polyclonal rabbit anti-human PTH1R antibody (antibody 1781; 0.1 μg/mL; Gramsch Laboratories, Schwabhausen, Germany), which was developed and extensively characterized by our group recently [9], a monoclonal mouse-anti-human OPG antibody (clone 5G2, Acris Antibodies, Herford, Germany; dilution, 1:100), or a mouse monoclonal anti-human RANKL antibody (clone 12A668; Enzo Life Sciences, Farmingdale, NY, USA; dilution, 1:750). Detection of the primary antibodies was performed using a biotinylated anti-rabbit or anti-mouse IgG, followed by incubation with peroxidase-conjugated avidin (Vector ABC “Elite” kit; Vector, Burlingame, CA, USA). Binding of the primary antibodies was visualized using 3-amino-9-ethylcarbazole in acetate buffer (BioGenex, San Ramon, CA, USA). Sections were then rinsed, counterstained with Mayer’s hematoxylin, and mounted in Vectamount® mounting medium (Vector Laboratories, Burlingame, CA, USA). Sections from human kidney (PTH1R, OPG), human placenta (OPG), and human duodenum (RANKL) served as positive controls. As negative control, the primary antibody was either omitted (OPG, RANKL, PTH1R) or adsorbed for 2 h at room temperature with 10 μg/ml of the peptide used for immunizations (PTH1R).

The proportion of trabecular structures within each bone sample was determined in the HE-stained samples at 100x magnification by means of the point counting method according to Bressot et al. [10] with slight modifications, using a 20 x 20 (1 cm x 1 cm) grid. Fifty visual fields were counted in each section, and an arithmetic mean was calculated.

Immunohistochemical stainings were scored using the semiquantitative Immunoreactivity Score (IRS) according to Remmele and Stegner [11], with separate scores calculated for osteoblasts, osteocytes, and osteoclasts. IRS was determined by multiplying the percentage of positive cells, stratified into five gradations (0, no positive cells; 1, <10% positive cells; 2, 10–50% positive cells; 3, 51–80% positive cells; 4, >80% positive cells), by the intensity of staining, stratified into four gradations (0, no staining; 1, mild staining; 2, moderate staining; 3, strong staining). IRS values ranged from 0 to 12. Decalcification time did not affect the immunohistochemical staining results (samples A: -0.054 < Spearman correlation coefficient (r_sp_) < 0.123; p > 0.206; samples B: -0.160 < r_sp_ < 0.098; p > 0.311). All immunohistochemical stainings were evaluated by two independent blinded investigators (JG, AL). In case of discrepant scores, final decision was achieved by consensus.

### Analysis of serum parameters

Serum levels of PTH, OPG, sRANKL, AP, osteocalcin, TRAP5b, TP1NP, ICTP, and sclerostin were determined using commercially available enzyme-linked immunosorbent assay (ELISA) kits according to the manufacturer’s instructions: PTH 1–84, human; OPG, human Quidel®; sRANKL, human, high sensitive; TRAP5b, MicroVue; Osteocalcin, MicroVue; Sclerostin (TECOmedical AG, Sissach, Switzerland); Alkaline Phosphatase ELISA Kit (Biotrend Chemikalien GmbH, Cologne, Germany); TP1NP (MyBioSource, San Diego, CA, USA); and human C-telopeptide of procollagen (ICTP) (Biozol, Eching, Germany). With the only exception of the AP kit, all kits contained low and high control sera, which were measured in parallel to the test samples. In case of AP, normal and pathological control sera were obtained from Biomed (Oberschleißheim, Germany) and used as positive controls.

### Statistics

The IBM SPSS statistics program version 22.0 (SPSS Inc., Chicago, IL, USA) was used for all analyses. Because the data were not normally distributed (according to the Kolmogorov-Smirnov test), these tests were used: Kruskal-Wallis, Mann-Whitney, Wilcoxon, Chi-square, Spearman’s rank correlation, and Kendall’s τ-b. A p value ≤ 0.05 was considered statistically significant. Data are shown as median and interquartile range or as arithmetic mean ± standard error of the mean (SEM).

## Results

### Patient characteristics

Samples were obtained from 49 men and 59 women, with differences in sex distribution between the four diagnosis groups (Table 1). In patients with coxarthrosis, female sex prevailed, whereas in the few patients with a hip fracture and dysplastic coxarthrosis, an approximately equal sex distribution was observed. Osteonecrosis was present only in men. The median patient age was 68 years overall (range, 37−89 years). The patients were distributed over the age groups as follows: 30−39 (n = 1), 40−49 (n = 7), 50−59 (n = 22), 60−69 (n = 26), 70−79 (n = 43), 80−89 (n = 9). The age was the highest in patients with a hip fracture and the lowest in patients with osteonecrosis and dysplastic coxarthrosis (Table 1).

**Table 1:** Patient characteristics (n = 108).

Diagnosis		Cox-arthrosis	Hip fracture	Osteo-necrosis	Hip dysplasia	All patients
**Sex**	male	41	2	2	4	49
**(number)**	female	51	3	0	5	59
	total	92	5	2	9	108
**Age**	median	70.0	81.0	50.5	55.0	68.0
**(years)**	mean	67.3	74.8	50.5	54.1	66.3

### Tissue and serum parameters of bone metabolism

#### Bone decalcification time and histologic parameters

**Decalcification time** (as a measure of bone mineralization and bone density) was significantly longer for samples A, which contained an additional layer of cartilage and cortical bone (median, 24 days; range, 3−228 days) than for samples B, which consisted mainly of cancellous bone (7 days; 3–99 days; Wilcoxon, p < 0.001). There was no correlation in the decalcification time between the samples A and B and no association between the decalcification time of the samples A or B and patient age or sex. There was no correlation between decalcification time and serum calcium or creatinine or GFR. In samples B, however, decalcification time was significantly shorter in patients with stage 3 renal insufficiency (mean, 6.20 ± 1.07 days) than in patients with stage 1 disease (11.61 ± 2.29 days) or stage 2 disease (11.49 ± 1.81 days; Mann-Whitney, p = 0.052 and 0.004, respectively).

**Fig 1:**
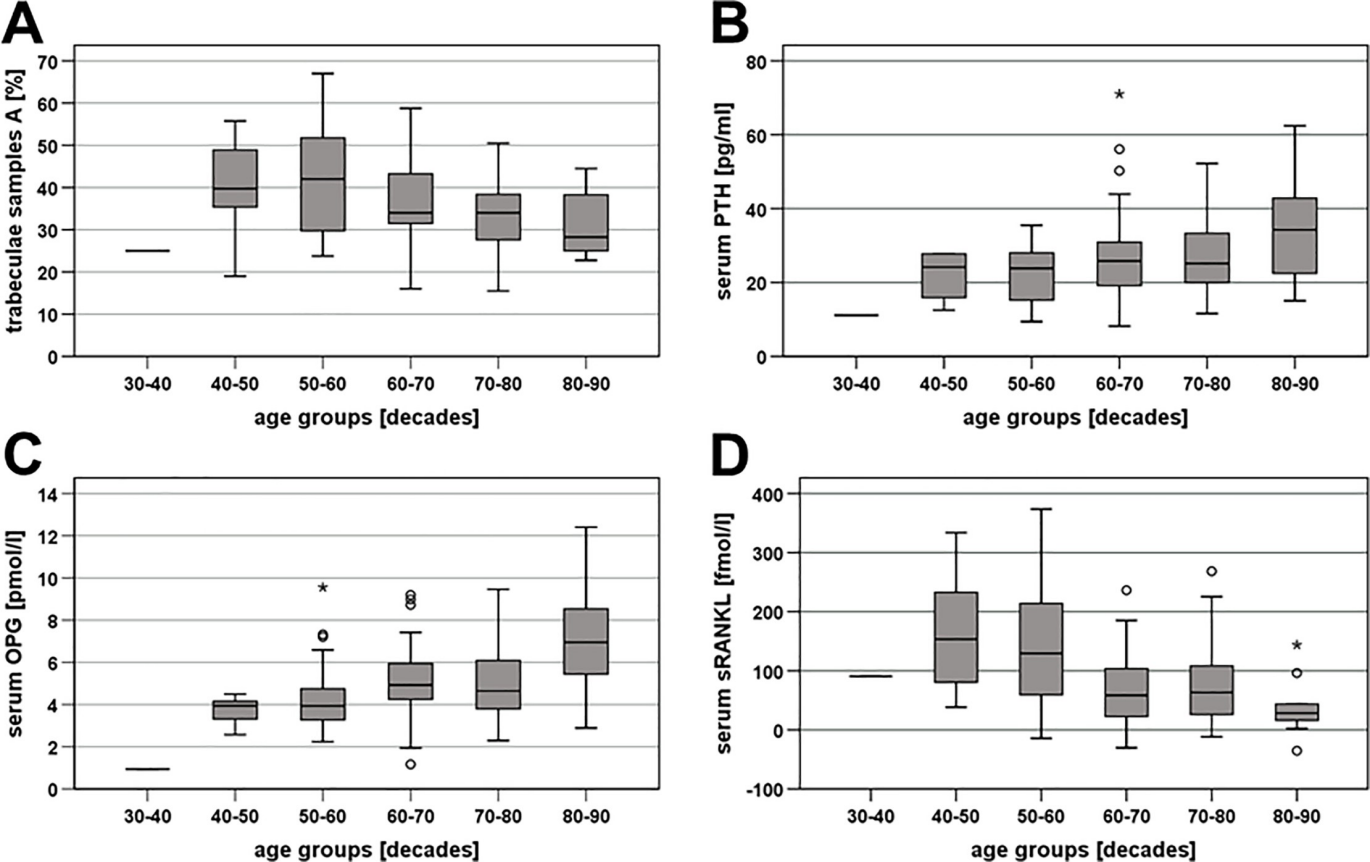
Influence of age on bone turnover markers. **A)** Percentage of trabecular mass in bone samples A, **B)** serum parathyroid hormone (PTH), **C)** serum osteoprotegerin (OPG), and **D)** serum soluble receptor activator of nuclear factor kappa-B ligand (sRANKL). Depicted are median values, upper and lower quartiles, minimum and maximum values, and outliers. The outliers are represented as circles for mild outliers (values 1.5–3 times above the upper quartile or below the lower quartile) and as asterisks for extreme outliers (data > 3 times above the upper quartile or below the lower quartile).

**Fig 2:**
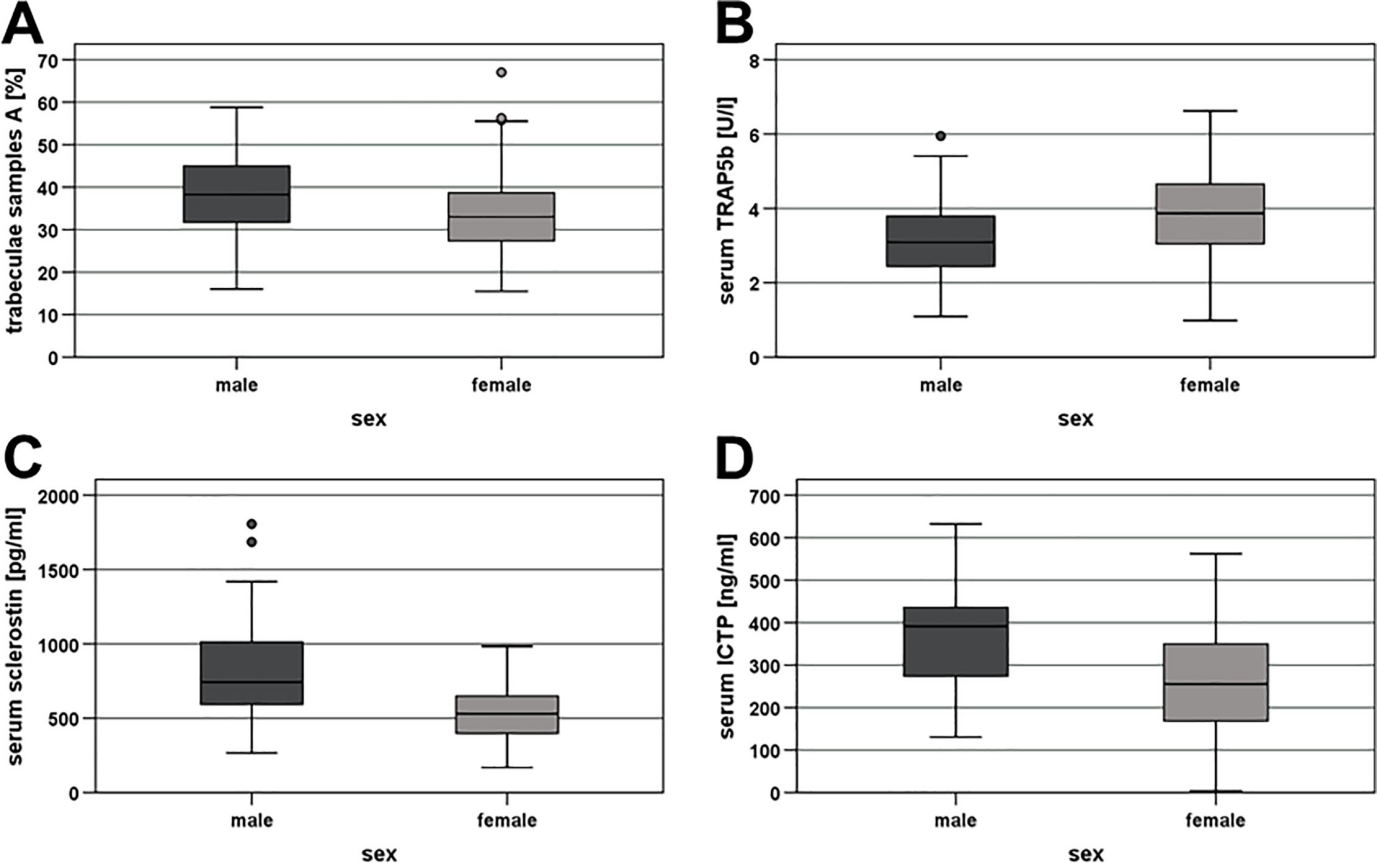
Influence of sex on bone turnover markers. **A)** Percentage of trabecular mass in bone samples A, **B)** serum tartrate-resistant acid phosphatase type 5b (TRAP5b), **C)** serum sclerostin, and **D)** serum C-telopeptide of type-1 collagen (ICTP). Depicted are median values, upper and lower quartiles, minimum and maximum values, and outliers. The outliers are represented as circles for mild outliers (values 1.5–3 times above the upper quartile or below the lower quartile) and as asterisks for extreme outliers (data > 3 times above the upper quartile or below the lower quartile).

**Fig 3:**
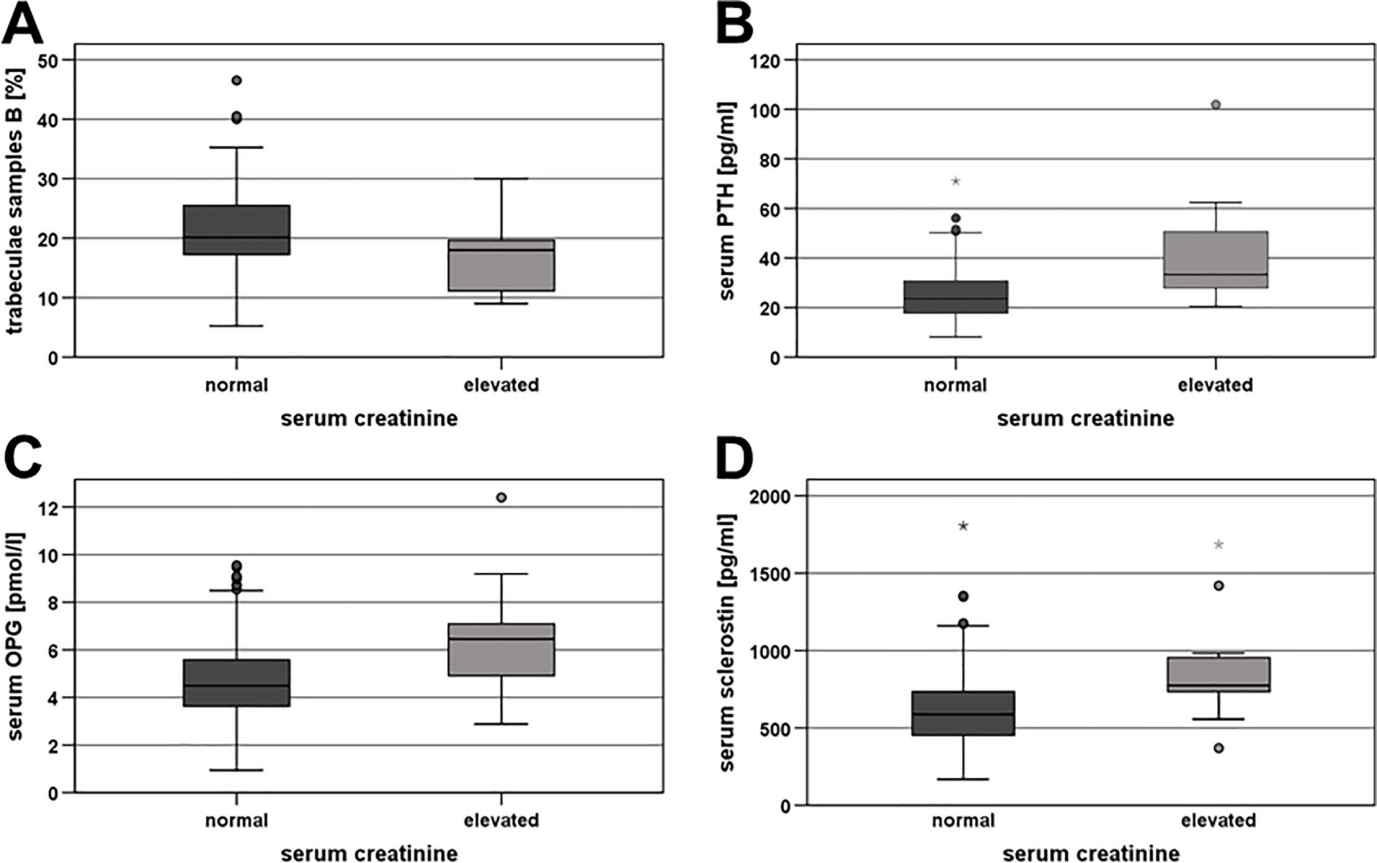
Influence of kidney function, as represented by normal or elevated serum creatinine, on bone turnover markers. Elevated creatinine values were defined as values > 97 μmol/L in women and > 106 μmol/L in men. **A)** Percentage of trabecular mass in bone samples B, **B)** serum parathyroid hormone (PTH), **C)** serum osteoprotegerin (OPG), and **D)** serum sclerostin. Depicted are median values, upper and lower quartiles, minimum and maximum values, and outliers. The outliers are represented as circles for mild outliers (values 1.5–3 times above the upper quartile or below the lower quartile) and as asterisks for extreme outliers (data > 3 times above the upper quartile or below the lower quartile).

The mean **proportion of trabecular bone** was 34.1% ± 10.4% in samples A and 21.3% ± 9.8% in samples B. Again, no correlation between the values of the samples A and B was observed. There was, however, a significant inverse correlation between the proportion of trabecular bone in samples A and patient age (r_sp_ = -0.196, p = 0.042; Fig 1A). Furthermore, in samples A, the proportion was significantly lower in women (mean, 34.4% ± 1.4%) than in men (38.3% ± 1.4%; Mann-Whitney, p = 0.021; Fig 2A). There was no correlation between the proportion of trabecular bone and serum calcium, but in samples B, the proportion was significantly lower in patients with an elevated creatinine (mean, 16.5% ± 1.8%) than in those with a normal creatinine (22.3% ± 1.0%; Mann-Whitney, p = 0.025; Fig 3A).

**PTH1R** was expressed on the plasma membrane of osteoblasts and osteocytes but not osteoclasts. Overall, PTH1R expression was very low in the bone samples investigated. It was significantly higher on osteoblasts (mean IRS, 2.77 ± 0.19) than on osteocytes (0.50 ± 0.11; Wilcoxon, p < 0.001) in samples A+B and significantly higher on osteoblasts in samples B (mean IRS, 3.23 ± 0.25) than on osteoblasts in samples A (2.31 ± 0.27; Wilcoxon, p = 0.007). Nevertheless, there was a significant correlation between PTH1R expression on osteoblasts and osteocytes (samples A, r_sp_ = 0.220, p = 0.022; samples B, r_sp_ = 0.196, p = 0.043; samples A+B, r_sp_ = 0.173, p = 0.073). In both samples A and B, PTH1R expression on osteoblasts and osteocytes was not associated with age, although the variability of PTH1R values increased with increasing age. PTH1R expression did not differ between men and women. Additionally, no association was observed between PTH1R expression and serum calcium or creatinine, GFR, or renal insufficiency stage.

Similar to PTH1R, **OPG** was only expressed on osteoblasts and osteocytes, but not on osteoclasts. Mean OPG IRS values were 4.69 ± 0.18 for osteoblasts and 4.37 ± 0.23 for osteocytes (in samples A+B), which were distinctly higher than the mean IRS values for PTH1R. There were no significant differences in OPG IRS values between samples A and B or between osteoblasts and osteocytes. However, a significant correlation between OPG expression on osteoblasts and osteocytes in samples A (r_sp_ = 0.375, p < 0.001) and A+B (r_sp_ = 0.273, p = 0.004) could be observed. Furthermore, OPG expression on osteocytes was negatively correlated with age (samples A+B, r_sp_ = -0.233, p = 0.015) but there was no difference between female and male patients. There was no association between bone OPG expression and serum calcium or creatinine, GFR, or renal insufficiency stage.

**RANKL** expression was likewise only detected on osteoblasts and osteocytes. Mean RANKL IRS values (samples A+B) were significantly higher in osteoblasts (4.25 ± 0.21) than in osteocytes (2.27 ± 0.15; Wilcoxon, p < 0.001). They were also significantly higher in osteoblasts and osteocytes of samples B than in the corresponding cells of samples A (Wilcoxon, p < 0.001). There was no association between bone RANKL expression and age, sex, or renal insufficiency stage. RANKL expression was also not correlated with serum calcium or creatinine or GFR.

#### Serum parameters

Mean **serum PTH** was 27.29 ± 1.31 pg/mL. While serum PTH was significantly correlated with age (r_sp_ = 0.288, p = 0.003; Fig 1B), it was not associated with sex or serum calcium. Serum PTH was, however, negatively correlated with GFR (r_sp_ = -0.259, p = 0.008) and positively associated with serum creatinine (r_sp_ = 0.202, p = 0.041). Accordingly, patients with an elevated creatinine or a reduced GFR had a significantly higher mean serum PTH (normal vs. elevated creatinine, 25.47 ± 1.17 pg/mL vs. 42.60 ± 7.07 pg/mL; Mann-Whitney, p = 0.018; Fig 3B; normal vs. reduced GFR, 25.63 ± 1.21 pg/mL vs. 36.53 ± 5.39 pg/mL; Mann-Whitney, p = 0.002).

**Serum OPG** was on average 5.12 ± 0.20 pmol/L. The values significantly correlated with age (r_sp_ = 0.292, p = 0.003; Fig 1C), but there was no difference between female and male patients. Furthermore, serum OPG was negatively correlated with serum calcium (r_sp_ = -0.213, p = 0.031). Although there was no association between serum OPG and absolute GFR or serum creatinine, there was a tendency towards higher mean OPG values in patients with an elevated serum creatinine (normal vs. elevated, 4.86 ± 0.20 pmol/L vs. 6.52 ± 0.79 pmol/L; Mann-Whitney, p = 0.053; Fig 3C) or reduced GFR (normal vs. reduced, 4.99 ± 0.22 pmol/L vs. 5.42 ± 0.57 pmol/L; Mann-Whitney, p = 0.082). Serum OPG values also differed according to stage of renal insufficiency (Kruskal-Wallis, p = 0.008). Here, OPG was significantly higher in patients with stage 3 renal insufficiency (mean, 6.81 ± 0.63 pmol/L) compared to those with stage 1 disease (4.75 ± 0.28 pmol/L) or stage 2 disease (4.78 ± 0.28 pmol/L; Mann-Whitney, p = 0.003 and 0.005, respectively).

Average **serum sRANKL** values amounted to 0.089 ± 0.008 pmol/l. The values negatively correlated with age (r_sp_ = -0.289, p = 0.003; Fig 1D) but not sex. There was no correlation between serum sRANKL and serum calcium or creatinine or GFR, but there was a tendency towards a lower sRANKL in patients with stage 3 renal insufficiency (mean, 0.0996 ± 0.0125 pmol/L) than in those with stage 1 disease (0.0654 ± 0.0236 pmol/L; Mann-Whitney, p = 0.056).

Mean **serum AP** was 32.74 ± 1.13 ng/mL. There was no association with age, sex, serum calcium or creatinine, GFR, or renal insufficiency stage.

Mean **serum osteocalcin** values were on average 0.695 ± 0.004 ng/mL. Osteocalcin was not associated with sex, serum calcium or creatinine, GFR, or stage of renal insufficiency. There was, however, a tendency towards a higher osteocalcin in women (mean, 0.702 ± 0.039 ng/mL) than in men (0.685 ± 0.046 ng/mL; Wilcoxon, p = 0.052).

Average **serum TP1NP** values were 32.74 ± 5.30 ng/mL. Again, no influence of patient age, sex, serum calcium or creatinine, GFR, or renal insufficiency stage was observed.

Mean **serum TRAP5b** was 3.66 ± 0.15 U/L. TRAP5b was not associated with age, serum calcium or creatinine, GFR, or stage of renal insufficiency. There was, however, a significant difference in TRAP5b between sexes (Mann-Whitney, p = 0.003), with a higher mean value in women (4.03 ± 0.23 U/L) than in men (3.22 ± 0.16 U/L; Fig 2B).

Average **serum sclerostin** values amounted to 0.662 ± 0.028 ng/mL. Values displayed a significant association with age (r_sp_ = 0.194; p = 0.048), as well as sex (Mann-Whitney, p < 0.001). In contrast to TRAP5b, sclerostin was lower in women (mean, 0.528 ± 0.023 ng/mL) than in men (0.832 ± 0.049 ng/mL; Fig 2C). Sclerostin was not associated with serum calcium. It was, however, positively correlated with serum creatinine (r_sp_ = 0.478, p < 0.001) and negatively associated with GFR (r_sp_ = -0.236, p = 0.016). Consequently, significantly higher sclerostin values were observed in patients with an elevated creatinine (mean, 0.883 ± 0.113 ng/mL) than in those with a normal creatinine (0.634 ± 0.029 ng/mL; Mann-Whitney, p = 0.008; Fig 3D). Sclerostin was also significantly higher in patients with stage 3 renal insufficiency (mean, 0.832 ± 0.096 ng/mL) compared to those with stage 1 disease (0.593 ± 0.035 ng/mL; Mann-Whitney, p = 0.022).

Mean **serum ICTP** was 311.78 ± 14.08 ng/mL. ICTP was not associated with age, serum calcium or creatinine, GFR, or renal insufficiency stage. As with sclerostin, ICTP was significantly lower in women (mean, 261.11 ± 18.58 ng/mL) than in men (364.54 ± 18.35 ng/mL; Mann-Whitney, p < 0.001; Fig 2D).

### Correlations between bone metabolism parameters

In both samples A and B, the **percentage of trabecular bone** was significantly correlated with decalcification time (samples A, r_sp_ = 0.422, p < 0.001; samples B, r_sp_ = 0.233; p < 0.001).

No correlation was found between **PTH1R expression** and decalcification time or percentage of trabecular bone. However, **OPG expression** on osteocytes in samples A was significantly associated with decalcification time (r_sp_ = 0.256, p = 0.008) and percentage of trabecular bone (r_sp_ = 0.220, p = 0.022) of these samples. Additionally, there was a significant correlation between OPG expression and PTH1R expression on osteoblasts plus osteocytes in samples A+B (r_sp_ = 0.283, p = 0.003), as well as on osteocytes in samples A+B (r_sp_ = 0.336, p < 0.001), but no significant correlation on osteoblasts alone in samples A+B (r_sp_ = 0.149, p = 0.124). Similar results were obtained when considering samples A and samples B separately.

**RANKL expression** on osteoblasts in samples A and samples A+B, as well as on osteocytes in samples A and on osteoblasts plus osteocytes in samples A+B, was negatively correlated with decalcification time (r_sp_ = -0.252, p = 0.008; r_sp_ = -0.201, p = 0.037; r_sp_ = -0.353, p < 0.001; r_sp_ = -0.258, p = 0.007, respectively). Additionally, significant negative associations were observed between the percentage of trabecular bone and RANKL expression on osteocytes in samples A (r_sp_ = -0.274, p = 0.004), on osteocytes in samples A+B (r_sp_ = -0.249, p = 0.009), and on osteoblasts plus osteocytes in samples A+B (r_sp_ = -0,213, p = 0.027). Besides, RANKL expression on osteoblasts in samples A was positively correlated with PTH1R expression on these cells (r_sp_ = 0.286, p = 0.003).

**Fig 4:**
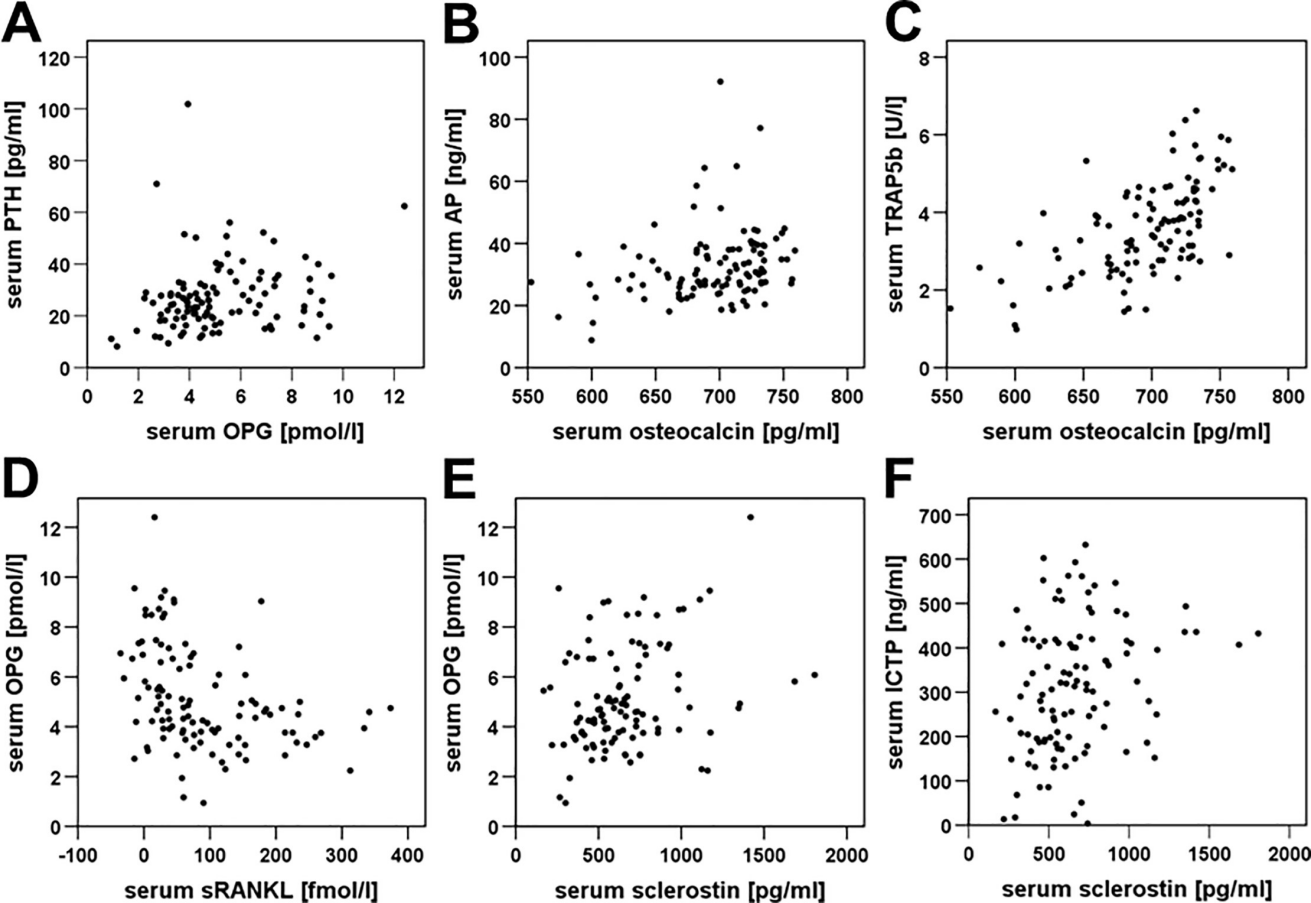
Correlations between serum bone turnover markers. **A)** Serum parathyroid hormone (PTH) vs. serum osteoprotegerin (OPG), **B)** serum alkaline phosphatase (AP) vs. serum osteocalcin, **C)** serum tartrate-resistant acid phosphatase type 5b (TRAP5b) vs. serum osteocalcin, **D)** serum OPG vs. serum soluble receptor activator of nuclear factor kappa-B ligand (sRANKL), **E)** serum OPG vs. serum sclerostin, and **F)** serum C-telopeptide of type-1 collagen (ICTP) vs. serum sclerostin. Data are presented as scatter plots.

No correlations were observed between serum PTH, OPG, AP, osteocalcin, or sclerostin and decalcification time, percentage of trabecular bone, or expression of PTH1R, OPG, or RANKL. There were, however, significant positive associations between **serum PTH** and **serum OPG** (r_sp_ = 0.268, p = 0.005; Table 2; Fig 4A), between serum PTH and **AP** (r_sp_ = 0.279, p = 0.003), and between serum OPG and AP (r_sp_ = 0.259, p = 0.007) (Table 2). A positive interrelationship was also observed between serum AP and **osteocalcin** (r_sp_ = 0.314, p = 0.001; Table 2; Fig 4B). **Serum TP1NP** showed a correlation with OPG expression on osteoblasts (r_sp_ = 0.195, p = 0.046), osteocytes (r_sp_ = 0.404, p < 0.001), and osteoblasts plus osteocytes (r_sp_ = 0.201, p = 0.039) in samples B as well as on osteoblasts plus osteocytes in samples A+B (r_sp_ = 0.236, p = 0.016). Additionally, a positive association with serum OPG was noticed (r_sp_ = 0.323, p = 0.001; Table 2). **Serum TRAP5b** was negatively correlated with trabecular mass in samples A (r_sp_ = -0.238, p = 0.014), but showed a positive association with serum AP (r_sp_ = 0.394, p < 0.001) and osteocalcin (r_sp_ = 0.596, p < 0.001; Table 2; Fig 4C). Regarding **sRANKL**, a negative association with PTH1R expression on osteocytes in samples A (r_sp_ = -0.200, p = 0.040) and samples A+B (r_sp_ = 0.222, p = 0.023) was observed, but no correlation with bone OPG or RANKL expression. There was also a negative association between sRANKL and serum OPG (r_sp_ = -0.432, p < 0.001; Table 2; Fig 4D) and a tendency towards a negative interrelationship between sRANKL and TP1NP (r_sp_ = -0.160, p = 0.098; Table 2). **Serum sclerostin** was positively correlated with serum OPG (r_sp_ = 0.254, p = 0.008; Table 2; Fig 4E) and exhibited also a tendency towards a positive association with serum PTH (r_sp_ = 0.186; p = 0.054). Between the bone resorption marker **ICTP** and trabecular mass in samples A a significant positive correlation (r_sp_ = 0.226, p = 0.020) was observed and a tendency towards an association between ICTP and trabecular mass in samples B (r_sp_ = 0.193, p = 0.062) and samples A+B (r_sp_ = 0.205, p = 0.051). There was also a positive correlation between serum ICTP and serum sclerostin (r_sp_ = 0.290, p = 0.002; Table 2; Fig 4F).

**Table 2:** Correlations between serum parameters.

parameter		PTH	OPG	AP	osteocalcin	TP1NP	TRAP5b	sRANKL	sclerostin	ICTP
**PTH**	**r**	---								
	**p**	---								
**OPG**	**r**	**0.268**	---							
	**p**	**0.005**	---							
**AP**	**r**	**0.279**	**0.259**	---						
	**p**	**0.003**	**0.007**	---						
**osteocalcin**	**r**	0.061	-0.042	**0.314**	---					
	**p**	0.530	0.664	**0.001**	---					
**TP1NP**	**r**	0.159	**0.323**	0.098	-0.102	---				
	**p**	0.100	**0.001**	0.312	0.294	---				
**TRAP5b**	**r**	-0.053	0.036	**0.394**	**0.596**	-0.030	---			
	**p**	0.588	0.712	**< 0.001**	**< 0.001**	0.758	---			
**sRANKL**	**r**	-0.058	**-0.432**	-0.092	0.105	-0.160	-0.122	---		
	**p**	0.554	**< 0.001**	0.346	0.281	0.098	0.208	---		
**sclerostin**	**r**	0.186	**0.254**	0.117	-0.009	0.078	-0.136	0.030	---	
	**p**	0.054	**0.008**	0.229	0.926	0.424	0.161	0.758	---	
**ICTP**	**r**	0.150	0.139	0.106	0.168	-0.008	-0.147	-0.100	**0.290**	---
	**p**	0.120	0.152	0.276	0.082	0.934	0.128	0.303	**0.002**	---

r, correlation coefficient; p, level of significance (Spearman). Significant correlations are indicated in bold.

AP, alkaline phosphatase; ICTP, C-telopeptide of type-1 collagen; OPG, osteoprotegerin; PTH, parathyroid hormone; sRANKL, soluble receptor activator of nuclear factor kappa B ligand; TP1NP, total procollagen type-1 intact N-terminal propeptide; TRAP5b, tartrate-resistant acid phosphatase type 5b.

## Discussion

### PTH1R, OPG, and RANKL expression in different bone cell populations

In the present study, PTH1R, OPG, and RANKL expression was observed not only on osteoblasts but also on osteocytes, corroborating recent recognition of the key role of osteocytes in bone turnover [12–14]. No PTH1R, OPG, or RANKL expression was detected on osteoclasts. While previous studies have been in agreement regarding the expression of PTH1R, OPG or RANKL on osteoblasts and osteocytes [5, 9, 12, 15–28], literature data are contradictory regarding their expression on osteoclasts. Whereas in some studies PTH1R, OPG or RANKL expression was detected also on osteoclasts [15, 21, 23, 29–31], in others this was not the case [9, 16, 17, 18, 22, 32, 33]. These discrepancies may be attributed not only to species or methodological differences but also to differences between normal and pathological conditions. For example, Carda et al. [23] detected OPG and RANKL expression on osteoclasts of the human embryonal craniomandibular joint when using immunohistochemistry, but not when using in-situ hybridization, and another study using in-situ hybridization revealed PTH1R expression only in actively resorbing osteoclasts under certain pathological conditions (e.g., hyperparathyroid bone, healing fracture callus, Pagetic bone), but not in normal human bone [19]. Additional studies are necessary for further clarification.

### Correlations between bone metabolism markers and clinical data

#### Patient age

The present study revealed a negative association between patient age of the whole cohort of patients and bone trabecular mass, reflecting the well-known increased risk of osteoporosis with increasing age [34–36]. This age-related decline in trabecular mass was paralleled by a decrease in bone OPG expression, whereas bone RANKL expression remained unchanged with age. These findings suggest that changes in OPG expression, leading to a secondary imbalance between OPG and RANKL expression, are predominantly responsible for bone loss in the ageing skeleton. This theory is corroborated by the observation that OPG-deficient mice develop early-onset osteoporosis [37]. Paradoxically, the age-dependent decline in bone trabecular mass and OPG expression was accompanied by an increase in serum PTH and OPG and a decrease in serum sRANKL. However, similar observations have been reported by numerous other authors (see e.g., [38–46]) and it has been suggested that these findings represent a compensatory response to progressive bone loss in aged individuals [47–49]. Besides in bone, OPG is produced by a variety of other organs and tissues including the cardiovascular system. Here, OPG has been associated with vascular calcification, artherosclerosis and cardiovascular mortality (which also increase with age) (see e.g., [50–56]). Therefore, the serum OPG values may also reflect the cardiovascular status of the patients. In the present study also for sclerostin a significant increase with age was observed. This marker is secreted primarily by osteocytes, has a negative effect on bone deposition, and has already been linked to age-related impairment of bone formation [57–59]. All other parameters assessed in the present study remained unchanged with increasing age. Of these, only TP1NP has been previously evaluated in this context and was also shown to be unaffected by age [59].

#### Patient sex

Compared with men, women exhibited less trabecular mass, lower serum sclerostin, and lower serum ICTP, but higher TRAP5b and a tendency towards higher osteocalcin. Except for serum sclerostin and ICTP, which have not been investigated in this context so far, these results correspond well to existing data in the literature [35, 36, 60–62]. They are consistent with the well-known higher risk of osteoporosis and lower total bone turnover in elderly women, compared with men, but point also to a higher number and activity of osteoclasts in women, as indicated by the higher TRAP5b levels. Because of their greater bone loss and according to the majority of the previous studies [40, 61–66], higher serum PTH and OPG levels would have been expected in female than in male patients. In the current study, PTH was similar in both sexes and OPG was somewhat higher in women than in men, although the difference did not reach statistical significance (mean OPG in women vs. men: 5.27 ± 1.99 vs. 4.77 ± 2.11; Mann-Whitney, p = 0.137).

#### Kidney function

In the present study, patients with impaired kidney function exhibited a shorter bone decalcification time (as an indirect measure for bone mineralization and bone density) and less bone trabecular mass, indicating the presence of renal osteopathy. Furthermore, in patients with kidney dysfunction lower sRANKL and higher serum PTH, OPG, and sclerostin were observed. These findings correspond well to the results of previous studies, which also observed lower bone mineral density [67], higher serum PTH [38, 46, 67–70], higher serum OPG [61, 62, 67, 68, 71, 72], and lower sRANKL [61, 62] in patients with renal insufficiency. These changes in serum PTH, OPG and sRANKL are likely compensatory to bone loss, similar to the changes associated with increasing age. In accordance with the findings in older individuals, sclerostin seems to be involved also in the pathogenesis of bone loss in renal osteopathy.

### Correlations between bone metabolism parameters

As expected, in the current study a positive correlation between bone decalcification time and trabecular mass was observed. There was also a positive association between OPG expression of the osteocytes of the samples A and the decalcification time of these samples as well as with the percentage of trabecular bone. These findings reflect the importance of osteocytes in bone metabolism, in addition to the protective role of OPG expression on bone mass. As expected, a negative correlation was observed between RANKL expression on both osteoblasts and osteocytes and bone decalcification time, as well as trabecular mass. However, there was also a positive relationship between PTH1R and OPG expression (mainly with osteocytes) and between PTH1R and RANKL expression (mainly with osteoblasts), reflecting the well-known dual role of PTH/PTH1R signalling in bone turnover (osteoanabolic or osteocatabolic, depending on the duration and periodicity of PTH action) [73].

Regarding serum parameters, positive correlations were noticed between serum PTH, OPG, and AP, all of which were upregulated with bone loss. These findings correspond well to data from the literature showing positive associations between PTH and AP [70, 74, 75] and between OPG and AP [47]. Additionally, and also consistent with previous results [74, 76, 77], a negative association was observed between serum OPG and sRANKL. This inverse relationship was expected since these parameters have opposing effects on bone turnover. Also as anticipated, in the current study positive correlations were observed between serum OPG and TP1NP, a marker for collagen production; between the two bone formation markers, AP and osteocalcin; and between the two bone resorption indicators, sclerostin and ICTP. Interestingly, there were also positive correlations between serum OPG and sclerostin; between serum AP and osteocalcin, both of which are secreted by osteoblasts; and between serum AP and TRAP5b, the latter of which is produced by osteoclasts. These findings indicate the presence of high bone turnover, which in many studies has been shown to be associated with an increased risk of osteoporosis and osteoporotic fractures [75, 78–82]. Previous studies have likewise reported correlations between OPG and TP1NP [64], between AP and TRAP5b [74], and between osteocalcin and TRAP5b [74]. Moreover, data from the literature suggest that, independent of bone mineral density, bone turnover markers may be useful for predicting osteoporotic fractures, with bone resorption markers having higher prognostic value than bone formation indicators [75, 79, 81, 82]. The present study also revealed a positive correlation between bone OPG expression and serum TN1NP and a negative association between trabecular mass and serum TRAP5b, both of which were expected.

Interestingly, the present study did not show associations between bone PTH1R expression and serum PTH, between bone OPG expression and serum OPG, or between bone RANKL expression and serum sRANKL. Similar findings have been previously reported for bone PTH1R and serum PTH [21] and for bone RANKL and serum sRANKL [27]. Thus, serum OPG or sRANKL are not useful indicators of bone OPG or RANKL expression. There also seems to be no direct effect of PTH on PTH1R expression, which may be attributed to the modulatory influences of other key factors in bone metabolism, as suggested by the observed correlations between bone PTH1R expression and OPG, as well as RANKL, expression. The lack of correlation between sRANKL values and bone RANKL expression may be explained by the existence of other sources of sRANKL in addition to bone cells (e.g., activated T lymphocytes) [83]. This is supported by our findings of a negative correlation between PTH1R expression and serum sRANKL and a positive association between PTH1R and RANKL expression.

### Conclusions

In the present study, a broad panel of tissue and serum parameters of bone metabolism was assessed in a large cohort of orthopaedic patients to evaluate possible relationships between these parameters and to correlate the results with clinical data. The study revealed a number of clear interrelationships between the tissue and serum bone metabolism markers investigated, which also increases the probability that the changes observed with these parameters are indeed related to bone metabolism. Additionally, the study identified characteristic changes in some of these parameters in dependence on age, sex, and kidney function, which makes them useful diagnostic or prognostic markers. Overall, these findings improve our understanding of bone metabolism under different physiological and pathological conditions, but further investigations with even larger groups of patients are necessary to validate the results.

## Abbreviations

AP: alkaline phosphatase
ICTP: C-telopeptide of type-1 collagen
IRS: Immunoreactivity Score
OPG: osteoprotegerin
PTH: parathyroid hormone
PTH1R: parathyroid hormone type 1 receptor
RANKL: receptor activator of nuclear factor kappa-B ligand
r_sp_: Spearman correlation coefficient
TB: trabecular bone
TP1NP: total procollagen type-1 intact N-terminal propeptide
TRAP5b: tartrate-resistant acid phosphatase type 5b
